# Integrated omics analyses reveal the details of metabolic adaptation of *Clostridium thermocellum* to lignocellulose-derived growth inhibitors released during the deconstruction of switchgrass

**DOI:** 10.1186/s13068-016-0697-5

**Published:** 2017-01-10

**Authors:** Suresh Poudel, Richard J. Giannone, Miguel Rodriguez, Babu Raman, Madhavi Z. Martin, Nancy L. Engle, Jonathan R. Mielenz, Intawat Nookaew, Steven D. Brown, Timothy J. Tschaplinski, David Ussery, Robert L. Hettich

**Affiliations:** 1 Biosciences Division, Oak Ridge National Lab, Oak Ridge, TN 37831 USA; 2 Chemical Sciences Division, Oak Ridge National Lab, Oak Ridge, TN 37831 USA; 3 Department of Genome Science and Technology, University of Tennessee, Knoxville, TN 37996 USA; 4 Dow AgroSciences, 9330 Zionsville Road, Indianapolis, IN 46268 USA; 5 Department of Biomedical Informatics, University of Arkansas for Medical Sciences, Little Rock, AR 72205 USA

**Keywords:** *Clostridium thermocellum*, Switchgrass, Lignocellulosic, Biofuel, Ethanol, Mass spectrometry, Proteomics, Metabolomics, Transcriptomics, Cellulosome

## Abstract

**Background:**

*Clostridium thermocellum* is capable of solubilizing and converting lignocellulosic biomass into ethanol. Although much of the work-to-date has centered on characterizing this microbe’s growth on model cellulosic substrates, such as cellobiose, Avicel, or filter paper, it is vitally important to understand its metabolism on more complex, lignocellulosic substrates to identify relevant industrial bottlenecks that could undermine efficient biofuel production. To this end, we have examined a time course progression of *C. thermocellum* grown on switchgrass to assess the metabolic and protein changes that occur during the conversion of plant biomass to ethanol.

**Results:**

The most striking feature of the metabolome was the observed accumulation of long-chain, branched fatty acids over time, implying an adaptive restructuring of *C. thermocellum’s* cellular membrane as the culture progresses. This is undoubtedly a response to the gradual accumulation of lignocellulose-derived inhibitory compounds as the organism deconstructs the switchgrass to access the embedded cellulose. Corroborating the metabolomics data, proteomic analysis revealed a corresponding time-dependent increase in various enzymes, including those involved in the interconversion of branched amino acids valine, leucine, and isoleucine to iso- and anteiso-fatty acid precursors. Additionally, the metabolic accumulation of hemicellulose-derived sugars and sugar alcohols concomitant with increased abundance of enzymes involved in C5 sugar metabolism/pentose phosphate pathway indicates that *C. thermocellum* shifts glycolytic intermediates to alternate pathways to modulate overall carbon flux in response to C5 sugar metabolites that increase during lignocellulose deconstruction.

**Conclusions:**

Integrated omic platforms provided complementary systems biological information that highlight *C. thermocellum*’s specific response to cytotoxic inhibitors released during the deconstruction and utilization of switchgrass. These additional viewpoints allowed us to fully realize the level to which the organism adapts to an increasingly challenging culture environment—information that will prove critical to *C. thermocellum*’s industrial efficacy.

**Electronic supplementary material:**

The online version of this article (doi:10.1186/s13068-016-0697-5) contains supplementary material, which is available to authorized users.

## Background

Switchgrass is a perennial, warm-season, C4 grass that is one of the dominant grasses in North America. It is a promising second-generation bioenergy feedstock due, in part, to its hardiness, high yields, low fertilizer requirements', and drought tolerance, and, thus, it has the potential to augment or replace existing starch-based processes for biofuel production [1]. Compared to first-generation biofuels, where added enzymes are used to deconstruct corn starch to dextrose for fermentation to ethanol by yeast, second-generation biofuels target the vast energy reserves stored in plant cell walls. Unlike starch, plant cell walls are generally difficult to deconstruct, since they consist of large, intertwined, recalcitrant biopolymers of C5 sugars (hemicellulose), C6 sugars (cellulose), and lignin [2, 3]. Accessing this reservoir of chemical energy requires the concerted action of multiple enzymes with diverse catalytic activities [4]—a bioengineering feat inherent to various cellulolytic microorganisms capable of solubilizing and ultimately consuming naturally abundant cell wall polysaccharides [5].

*Clostridium thermocellum* is an industrially relevant, cellulolytic microbe that efficiently deconstructs lignocellulosic biomass into sugars, which are fermented into ethanol and other products. As an anaerobic thermophile, this Gram-positive bacterium can be found in natural environments where cellulose degradation actively occurs (e.g., compost piles). It produces large extracellular enzyme complexes called cellulosomes that are predominantly tethered to the cell surface but can exist as free entities, enabling the efficient solubilization and deconstruction of lignocellulose to simpler sugars [6, 7]. Paired with the organism’s innate ability to ferment sugar to ethanol, the presence of cellulosomes makes *C. thermocellum*, an ideal candidate for consolidated bioprocessing (CBP), a “one-pot” industrial process whereby lignocellulosic biomass is converted directly into biofuel [8].

The cellulosome, replete with feedstock-optimized carbohydrate-active enzymes (CAZymes) [9], directs the conversion of cellulose to small, importable cellodextrins [10]. Intracellularly, these cellodextrins are further broken down into cellobiose and finally glucose, which is ultimately utilized by the organism to generate energy via fermentation to ethanol, acetic acid, lactic acid, hydrogen, and/or carbon dioxide [10]. From a bioethanol perspective, the generation of lactate, formate, and acetate remain undesirable as these competing metabolic pathways divert carbon flux away from ethanol and create a less hospitable environment when the organism is confined to culture/industrial fermenters. Although recent efforts to maximize ethanol yield in *C. thermocellum* by knocking out competing pathways has substantially increased the titer of ethanol produced, [11] much of the work-to-date focused on optimizing cellulose conversion to ethanol with model substrates, such as cellobiose, filter paper, and/or Avicel [12–15]. Thus, studies examining bacterial growth on more complex, recalcitrant, lignocellulosic material are essential, especially as the deconstruction of natural biomass is known to generate numerous antimicrobial and/or phenolic inhibitors that could ultimately impede the industrial process [16, 17].

There are few studies to date investigating *C. thermocellum*’s systematic response to the growth on bioenergy-relevant, lignocellulosic biomass such as pretreated switchgrass and/or *Populus* [18, 19]. These studies provided important clues as to how *C. thermocellum* deconstructs lignocellulosic biomass, but focused solely on gene expression and did not examine growth-dependent protein machinery nor the accumulation of important metabolites that could better inform the highly coordinated enzymatic process. To this end, we sought to formulate a more comprehensive, systems biology view of the deconstruction and conversion of switchgrass to ethanol by *C. thermocellum* over the course of batch fermentation. By integrating data obtained from three omic platforms—LC–MS/MS-based shotgun proteomics, microarray-based transcription profiling, and GC–MS-based metabolomics—we were detailed the mechanisms by which *C. thermocellum* adapts to the adverse environment created during lignocellulosic deconstruction, namely the release of switchgrass-derived compounds inhibitory toward growth.

To our knowledge, this is the first integrated omics interrogation of *C. thermocellum*’s deconstruction of a bioenergy-relevant feedstock. As the organism converts released sugars to a myriad of products, it must avoid and/or ameliorate the effects of both product-inhibition and biomass-derived cytotoxic metabolites. This information will be vitally important to metabolic engineering efforts that aim to enhance the industrial viability of bioethanol and other specialty biofuels/bioproducts.

## Methods

### Cultivation and sampling

Inoculum and triplicate fermentations of *C. thermocellum* ATCC 27405 were performed in 5-l Twin BIOSTAT^®^ B fermenters (Sartorius Stedim North America, Bohemia, NY), as previously described, except that all vessels contained 10 g/l (dry weight basis) dilute acid pretreated switchgrass as the main substrate [18]. Switchgrass from 4-year-old plants was pretreated with dilute sulfuric acid at the National Renewable Energy Laboratory (NREL, Golden, Colorado, USA), as previously described [18], washed several times with deionized water to remove soluble sugars, and dried overnight at 45 °C. MTC media were sparged overnight with nitrogen (to insure that the system was anaerobic and ready for *C. thermocellum* growth) before inoculation (10% v/v inoculum) to a final volume of 4 l, and the growth temperature was maintained at 58 °C [20]. The pH was controlled at 7.0 in the fermenters with 3 N NaOH (see Additional file 1: Text S1 for additional details on fermentation). Samples were collected for metabolomics and proteomics at 19-, 43-, 91-, and 187-h postinoculation. Samples for transcriptomics were collected at 19 and 43 h. Microarray data and platform details have been deposited in the NCBI Gene Expression Omnibus (GEO) database under accession number GSE26926, with data used in this study having accession numbers GSM663002-GSM663007.

### Metabolomic measurements

*Clostridium thermocellum* switchgrass fermentation samples were measured at 19, 43, 91, and 187 h, as matched for proteomic samples. Frozen cell pellets containing both microbe and plant material were weighed into 50 ml centrifuge tubes containing 10 ml of 80% ethanol, and 50 µl sorbitol (0.01000 g/ml) added as an internal standard. Samples were sonicated for 5 min (30 s on, 30 s off with an amplitude of 30%) and kept on ice. Samples were then centrifuged at 4500 rpm for 20 min, and the supernatant was decanted into scintillation vials and stored at −20 °C. One milliliter per sample was dried down, dissolved in 0.5 ml acetonitrile, and silylated to generate trimethylsilyl derivatives, as described elsewhere [21]. After 2 days, 1 µl aliquots were injected into an Agilent 5975C inert XL gas chromatograph–mass spectrometer (GC–MS). The standard quadrupole GC–MS was operated in the electron impact (70 eV) ionization mode, targeting 2.5 full-spectrum (50–650 Da) scans per second, as described previously [21] (see Additional file 1: Text S1 for additional details regarding metabolites quantification). Metabolite data were expressed as fold change relative to the 19-h sampling time point with significant differences determined with Student’s *t* tests. Significant differences in fold change between sampling time points were also assessed with Student’s *t* tests.

### Proteomic measurements

50 ml fermentation samples were centrifuged at 8000 rpm for 20 min in a Sorvall Legend RT centrifuge (ThermoScientific, Waltham, MA, USA), and the supernatant was decanted, leaving a 5 ml pellet comprised SWG biomass, *C. thermocellum*, and substrate-bound cellulosomes, all of which were flash frozen in liquid nitrogen. The composite pellet was resuspended in 6 M guanidine-HCl, 10 mM DTT, and pH 8.0, and cells were then lysed by sonic disruption (*Branson* Ultrasonics Corp., Danbury, CT, USA). The resulting crude lysates were processed for trypsin-based bottom-up proteomics, as described in Additional file 1: Text S1. One hundred micrograms of tryptic peptides loaded onto a MudPIT column and analyzed over the course of 24-h via data dependent acquisition on a LTQ-XL mass spectrometer (Thermo Scientific). See Additional file 1: Text S1 for specific details regarding the LC separation and mapping of MS/MS spectra to predicted *C. thermocellum* peptides.

Prior to semi-quantitative analysis, spectral counts were rebalanced to properly distribute non-unique/shared peptides between their potential parent proteins, as previously described [22], and raw SpC values were converted to normalized spectral abundance factors (NSAF) [23] to assess quantitative differences between time points. The normalized counts of individual proteins were statistically evaluated across different time points of growth using *p* values derived from one-way ANOVA. Proteins with *p* values <0.05 were used for further clustering analysis. C-mean clustering of the proteins through Mfuzz [24] package was performed to identify common temporal responses. Functional enrichment (COG, KEGG, and GO) of individual cluster were performed using Fisher’s exact test through PIANO [25] package to generate a subnetwork of functional terms based on overlap members using a *p* value <0.01 in Cytoscape version 3.2.1. *C. thermocellum* metabolic pathways information were obtained from the KEGG pathway database [26]. Transcriptomics measurements (19 and 43 h) and prediction of highly expressed regions of the DNA were integrated with the proteomics data and presented as a genome atlas (Fig. 2). The details of the transcriptomic measurements, as well as prediction of highly expressed genes using ‘positional preference,’ are described in Additional file 1: Text S1. All raw mass spectra for the proteome measurements have been deposited into the ProteomeXchange repository with the following accession numbers: ProteomeXchange: PXD004905; MassIVE: MSV000080128) and is available at ftp://MSV000080128@massive.ucsd.edu [password: swgtc (for reviewer access)].

## Results and discussion

### Microbial growth characteristics on switchgrass

**Fig. 1:**
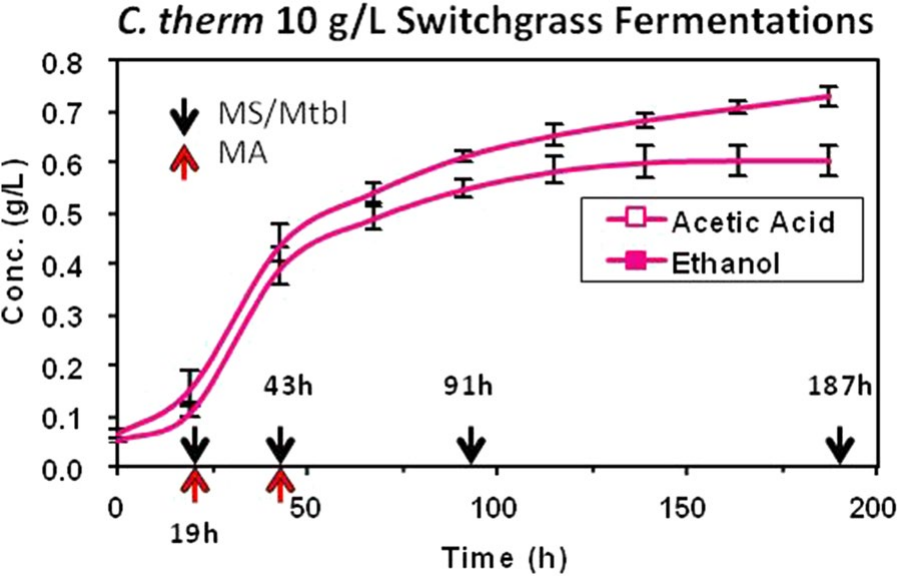
Concentration (g/l) of acetic acid and ethanol (data reported as the average from triplicate fermentations on pretreated switchgrass). *Arrows* indicate the sampling points used for mass spectrometry (MS), metabolomics (Mtbl), and microarray (MA)

Acetic acid and ethanol were the major fermentation products for wild-type *C. thermocellum* grown on pretreated switchgrass (Fig. 1). *C. thermocellum* derives ATP coupled with the production of acetic acid and ethanol. Thus, both are indicative of metabolic activity and cell growth. However, acetic acid can also be generated through deacetylation of hemicelluloses, [27] and thus the relative contributions from the pretreated biomass or bacteria cannot be easily differentiated in this study. Ethanol production, however, is exclusive to *C. thermocellum* and therefore a more specific indicator of microbial growth, especially given the complex culture conditions. The trends for acetic acid and ethanol were similar until 120-h postinoculation. Although the concentration of acetic acid was found to be 0.6 g/l after 139 h of microbial growth, ethanol stopped accumulating after 120 h. Acetic acid increased from ≤0.075 g/l at time zero to ~0.7 g/l after 187 h, and it continued to slowly accumulate throughout the experiment, consistent with other studies [27]. The time points of 19 and 43 h represent early and late exponential growth phase, respectively, and the cultures began to transition into stationary phase around 100-h postinoculation. Earlier fermentations with this wild-type strain and substrate produced 0.5 and 0.2 g/l of acetic acid and ethanol, respectively, after 37 h [18]. As final ethanol and acetic acid concentrations are low, the observed metabolic adaptations described below are likely in response to the chronic accumulation of other switchgrass-derived cytotoxic compounds that impact overall organismal growth.

### Metabolomic analysis indicates accumulation of key metabolites that may be inhibitory to *C. thermocellum* growth on lignocellulosic biomass

Metabolomic analysis provided the basis for a broad analysis of metabolites resulting from *C. thermocellum’*s deconstruction of growth on complex lignocellulosic biomass and revealed the accumulation of metabolites derived from hemicellulose, including xylobiose, xylose, xylitol, arabinose, and arabitol (Additional file 2: Table S1). Although dilute acid pretreatment is expected to remove much of the hemicellulose, especially xylan polymers, [28] metabolomic identification of depolymerized xylan products suggests that a measurable portion remains even after extensive washing. This is substantiated by the continued accumulation of metabolites such as xylose and xylobiose over time, which are indicative of continued enzymatic hydrolysis. Metabolite responses are presented as the ratios of metabolite concentration at different time points (43, 91, and 187 h) relative to 19 h, the first sampling point. Of these metabolites, xylitol exhibited the largest accumulation (~9-fold) as the culture reached stationary phase. Other 5-carbon sugar alcohols, such as arabitol and ribitol, and phenolic acids derived from lignin, including caffeic acid, ferulic acid and *p*-coumaric acid also accumulated. A previous study of lignocellulosic hydrolysates found that many of the hemicellulose and lignin-derived components are inhibitory toward microbial growth [29]. Thus, one must consider these cytotoxic compounds, since sugar alcohols, such as arabitol, have been found to be inhibitory to other microbes, such as *T. accharolyticum* (Raman B., Tschaplinski T.J., personal communication). This is especially relevant as wild-type *C. thermocellum* strains do not utilize hemicellulose-derived sugars.

In addition to biomass-derived metabolites, microbial-based fatty acid synthesis was evident throughout the course of the experiment. Isopalmitic acid (C16), isostearic acid (C18), and palmityl palmitic acid (C32) increased by ~15-fold by stationary phase. Other odd-carbon fatty acids, such as hentriancontanoic acid (C_31_H_62_O_2_), nonacosanoic acid (C_29_H_58_O_2_), heptacosanoic acid (C_27_H_54_O), and the methyl-branched iso-heptadecanoic acid (iso-C_17_H_34_O_2_), exhibited similar accumulation patterns, the latter of which increased by almost 21-fold. Since these are higher order and have odd numbers of carbon atoms, they are likely of microbial origin [30]. The accumulation of these long-chain, saturated fatty acids, and unique branched fatty acids, indicates cell membrane restructuring to better tolerate increased concentrations of lignocellulose-derived inhibitors that affect membrane fluidity [31–33].

These data indicate that the growth of *C. thermocellum* on switchgrass is a complicated process whereby plant-derived glucans, which are not limiting here (Additional file 2: Table S1), fuel microbial growth, and further drive biomass deconstruction, invariably leading to the accumulation of inhibitory metabolites that negatively impact microbial metabolism. Without the active removal/bioconversion of these inhibitory compounds and/or increased tolerance, the potential application of this cellulolytic organism for effective biofuel production will be limited, especially considering the planned industrial use of complex lignocellulosic substrates like switchgrass. Fortunately, studies have shown that it is possible to generate *C. thermocellum* strains with improved tolerance to complex lignocellulosic substrates, such as *Populus* hydrolysates [34].

### Proteomic and transcriptomic analyses reveal details of switchgrass-induced gene regulation

We extended our analyses to examine gene expression as it relates to the temporal dynamics of the proteome to better understand how *C. thermocellum* metabolism changes during growth and deconstructive/fermentative inhibition on switchgrass. Overall, 1551 unique proteins were identified in this study, representing ~50% of the predicted proteins listed in the *C. thermocellum* 27405 genome (Additional file 3: Table S2), in accord with other proteome measurements of *C. thermocellum* grown on Avicel or cellobiose [12, 35]. This provided reasonable coverage of key *C. thermocellum* systems (Additional file 4: Table S3) and allowed for detailed quantitative comparisons across the distinct lag/early exponential, late exponential and stationary growth phases to identify major proteins and pathways involved both the bioconversion of cellodextrins to energy and ethanol as well as the organism’s response to lignocellulosic metabolites released over the course of switchgrass deconstruction.

**Fig. 2:**
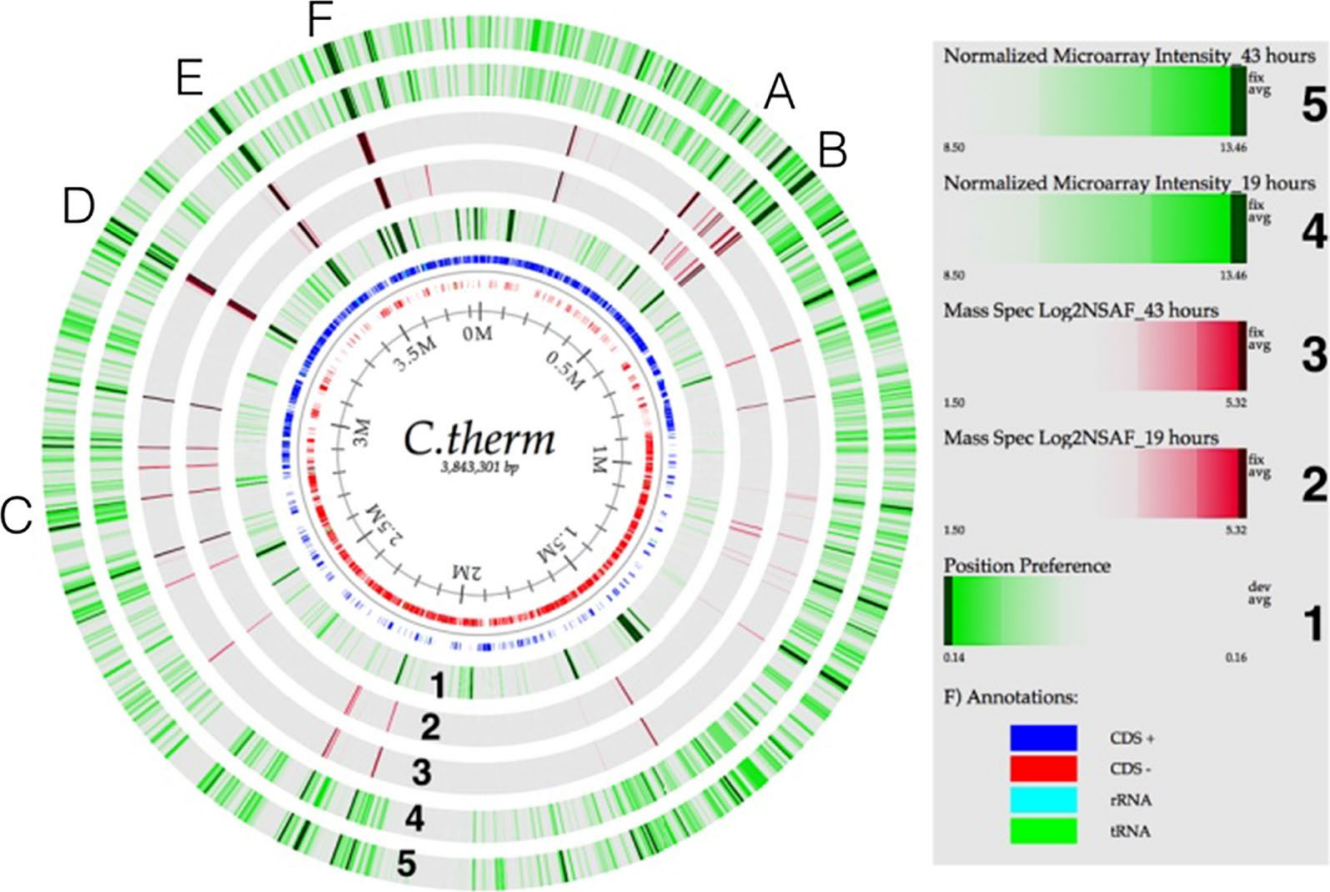
Genome atlas showing predicted highly expressed genes based on position preference (PP), log2(NSAF), and log2(Intensity) of microarray at time points 19 and 43 h. The innermost circle with bases position is the circular genome. *Inner circular lane* shows the position preference measure. The *next two lanes* represent mass spectrometry results (*inner lanes*) and microarray results (*outer lane*s) for time point 19 and 43 h. Regions A–F are marked in the figure. Details of genes and their location of these regions are in Additional file 6: Table S5

From a metabolic engineering standpoint, understanding the relationship between gene transcription (mRNA) and translation (protein) is valuable [36]. However, insufficient RNA quality was achieved for later two time points (93 and 187 h) that precluded microarray measurements for those two samples. Nevertheless, RNA measurements from two early time points (19 and 43 h) were used to compare to similar temporal proteome measurements. Since these two transcriptomics time points correspond to early and late exponential phase, respectively, these likely reflect the most significant physiological changes in the most active growth stages. With regard to gene expression, transcriptomic measurements revealed 435 genes with varied expression (*p* < 0.05; fold change > or < 2.0). Among these differentially expressed genes, 38 were significantly up-regulated, while 397 were significantly down-regulated (Additional file 5: Table S4) between the time points 19 and 43 h. From a fundamental standpoint, it is possible to predict regions in the chromosome that are likely to contain genes that can be highly expressed, based on chromatin accessibility [37, 38]. Analysis of the *C. thermocellum* ATCC 27405 genome revealed 29 such regions along the chromosome, as indicated by dark green regions in the ‘position preference’ lane in Fig. 2. We identified 84 genes located in these regions that are less likely to be condensed by chromatin proteins and thus should be highly expressed under the appropriate conditions. Among these 84 genes, 58 (~68%) proteins expressed from these genes were detected by mass spectrometry. The comparison table of position preference, gene expression, and MS detected proteins is given in Additional file 6: Table S5. This information is a useful resource for identifying promoter sequences, open and silent regions of the genome for targeted genetics/metabolic engineering, and importantly, identification of novel metabolic features important for growth on industrially relevant substrates.

In general, the correlations of transcripts with proteins in the *C. thermocellum* genome are illustrated with the red/green darkly shaded regions in the outer circles. There are six regions along the chromosome (marked A–F in Fig. 2) that contain the top set of expressed genes and their abundant proteins (35 proteins see Additional file 6: Table S5). Region B encodes CelK (Cthe_0412) and CbhA (Cthe_0413), which belong to glycoside hydrolase family 9 and are both involved in polysaccharide catabolic processing. Region F encodes CipA (Cthe_3077), OlpB (Cthe_3078), and cell surface glycoprotein 2 (Orf2p; Cthe_3079), which are essential, non-catalytic cellulosome anchoring proteins. Regions A, C, D, and E encode various protein products, including NADH dehydrogenase, ferredoxin, S-layer homology domain, ribosomal proteins, DNA-directed RNA-polymerase beta subunit, and adenylate kinases [38]. These regions are likely highly expressed under most growth conditions, but obviously can be regulated under specific growth conditions. This is exemplified by regions predicted to be highly expressed, but were detected at lower abundance (protein) or expression (mRNA) via mass spectrometry or microarray, respectively (Additional file 6: Table S5). These may be growth condition-dependent factors that are not employed for switchgrass solubilization.

### Clustering of protein abundance highlights major temporal trend patterns and their overall functional signatures

Using the proteome data for relative quantitation, we sought to investigate the temporal abundance patterns of proteins during microbial growth on switchgrass. One-way ANOVA revealed 566 proteins that were significantly time dependent (*p* < 0.05; Additional file 7: Table S6), with each falling into one of four clusters based on the similarities between their temporal abundance profiles (Additional file 8: Fig. S1a–d). Proteins belonging to cluster 1 show a gradual decrease until late exponential phase (91 h), followed by a sharp decrease in protein abundance toward the late-stationary phase. Cluster 2 represents proteins that gradually decrease in abundance throughout the entire fermentation process. Cluster 3 represents proteins that generally increase over time, and cluster 4 represents proteins that are growth phase dependent, i.e., those that remain relatively unchanged between exponential phase time points (19 and 43 h) with a sharp increase at early log (91 h) followed by an almost negligible increase toward the end of culture (187 h).

In order to examine differential protein expression at a broad functional level, proteins within each cluster were further classified based on their COG classification, KEGG pathways, and GO categories. Functional enrichment (Fisher’s exact test) was performed on all known annotations to identify statistically significant (*p* < 0.01) under- or over-represented functional categories. Cluster 1 is comprised primarily of ribosomal proteins, translation-related proteins, and proteins involved in ribosome biogenesis (Additional file 8: Fig. S1a)—processes related to overall microbial growth. Thus, the precipitous fall in abundance toward late-stationary would be expected due to overall cessation of growth and metabolic activity. Cluster 2 contains some ribosome-related proteins, proteins involved in 2-oxocarboxylic acid metabolism, biosynthesis of amino acids (valine, leucine, and isoleucine), glycolysis, and TCA cycle (Additional file 8: Fig. S1b). Functional enrichment of cluster 3 shows evidence for endo-1,4-beta-xylanase activity, carbohydrate transport and metabolism, and polysaccharide catabolic processes (Additional file 8: Fig. S1c), while cluster 4 represents cellulose catabolism, post translational modification, cell division, S-layer, bacterial secretion system, ATP binding, protein export, and intracellular trafficking and secretion (Additional file 8: Fig. S1d). The predominant members of cluster 3 and cluster 4, which both generally show increased protein abundance over time, are cellulosomal proteins, which will be discussed in detail below.

### Cellulosomal and other carbohydrate-active enzymes initiate cellulose deconstruction and solubilization

The first step toward switchgrass utilization by *C. thermocellum* involves solubilization of the complex lignocellulosic material by the organism’s cellulosome, and to a lesser extent its free enzyme systems [39]. Cellulosomes are large, extracellular protein complexes, comprised a multitude of enzymes tethered to the cell surface through scaffold-like anchoring proteins [10]. Overall, cellulosomal proteins exhibited increased abundance over time, with 30 of 32 significantly changing cellulosome components belonging to clusters 3 or 4 (Table 1). Four structural proteins called scaffoldins (CipA, OlpB, Orf2p, and OlpA) were among the most abundant of all detected cellulosomal proteins and were exclusive to cluster 4, which is consistent with other studies [4]. Interestingly, two other scaffoldins, SbdA and OlpC, decreased significantly with time (cluster 2). Aside from CipA, the main enzymatic scaffold of the cellulosome, the above listed proteins are cell surface associated and thus involved in anchoring cellulosomal components to *C. thermocellum*. OlpA and OlpC essentially bind one catalytic enzyme each, while the other three bind CipA. CipA itself contains nine type I cohesin domains that are populated by a myriad of interchangeable, catalytic CAZymes. SdbA, Orf2p, and OlpB bind 1, 2, or 7 CipA scaffolds, respectively, leading to multiplicative increases in the number of catalytic enzymes decorating *C. thermocellum* (i.e., 9, 18, or 63 catalytic enzymes, respectively) [7]. Of the three, only SdbA (1 CipA, 9 enzymes) decreases over time, indicating the decreased importance of this cell surface associate protein as the culture progresses. This is especially relevant as the other two can be “loaded” with relatively more catalytic enzyme, suggesting enhanced rates of local cellulose hydrolysis may be required as the culture progresses, perhaps as *C. thermocellum* begins to colonize the solid switchgrass substrate.

**Table 1:** Cellulosomal and non-cellulosomal carbohydrate-active enzymes showing significant temporal dynamics

Locus tag	Protein name	Protein function	19 h	43 h	91 h	187 h	*p* value (<0.05)	Cluster
Non-catalytic cellulosomal proteins								
Cthe_3079	Orf2p	Cellulosome anchoring protein, cohesin region	5.18	5.57	6.46	6.99	2.27E−05	C4
Cthe_0452	OlpC	Cellulosome anchoring protein, cohesin region	2.32	–1.06	–0.76	–0.58	1.41E−04	C2
Cthe_3077	CipA	Cellulosome anchoring protein, cohesin region	9.61	9.81	10.14	10.6	5.32E−04	C4
Cthe_3080	OlpA	Cellulosome anchoring protein, cohesin region	5.73	6.51	7.17	7.44	4.01E−03	C4
Cthe_1307	SdbA	Cellulosome anchoring protein, cohesin region	4.92	4.06	4.08	3.84	8.68E−03	C2
Cthe_3078	OlpB	Cellulosome anchoring protein, cohesin region	7.4	7.66	8.17	8.02	1.24E−02	C4
Cellulosomal cellulase genes								
Cthe_0044	CseP	Cellulosome enzyme, dockerin type I	–1.94	–2.12	0.12	2.68	4.69E−04	C3
Cthe_2972	XynA	Glycoside hydrolase, family 11	3.96	4.44	5.74	6.71	4.86E−04	C3
Cthe_0912	XynY	Glycoside hydrolase, family 10	–1.36	–3.06	0.54	1.98	5.88E−04	C3
Cthe_3132	Cthe_3132	Cellulosome enzyme, dockerin type I	–1.56	0.49	1.92	2.88	6.80E−04	C3
Cthe_1963	XynZ	Glycoside hydrolase, family 10	2.39	2.63	3.75	4.73	9.20E−04	C3
Cthe_2147	CelO	Glycoside hydrolase, family 5	–2.25	−2.4	0.81	1.54	2.05E−03	C3
Cthe_0239	Cthe_0239	Cellulosome enzyme, dockerin type I	1.43	1.82	2.7	3.56	2.10E−03	C3
Cthe_0745	CelW	Glycoside hydrolase, family 9	0.23	1.11	2.14	2.16	2.47E−03	C3
Cthe_1472	CelH	Carbohydrate binding family 11	–0.69	–2.18	0.23	1.32	3.22E−03	C3
Cthe_0797	CelE	Glycoside hydrolase, family 5	2.46	2.4	3.05	3.91	9.96E−03	C4
Cthe_2590	XynD	Glycoside hydrolase, family 10	−0.6	–0.27	1.7	2.65	1.06E−02	C3
Cthe_3141	Cthe_3141	Lipolytic enzyme, G-D-S-L	0.14	–0.36	1.96	2.58	1.12E−02	C3
Cthe_0433	Cthe_0433	Glycoside hydrolase, family 9	2.41	1.08	2.63	3.49	1.41E−02	C4
Cthe_0043	CelN	Glycoside hydrolase, family 9	1.37	1.64	2.35	2.91	1.55E−02	C4
Cthe_1806	Cthe_1806	Cellulosome enzyme, dockerin type I	1.55	1.1	2.22	2.8	1.81E−02	C4
Cthe_0625	CelQ	Glycoside hydrolase, family 9	2.5	1.46	2.29	2.43	1.93E−02	C4
Cthe_0274	CelP	Glycoside hydrolase, family 9	1.59	1.88	2.68	3.11	2.68E−02	C3
Cthe_0190	Cthe_0190	Proteinase inhibitor I4, serpin	1.47	2.05	3.57	4.36	3.15E−02	C3
Cthe_0258	Doc258	Cellulosome enzyme, dockerin type I	2.58	2.29	3.52	3.82	3.92E−02	C4
Free enzyme system								
Cthe_2809	LicA	Glycoside hydrolase, family 16	3.53	4.03	4.9	5.32	8.76E−04	C4
Cthe_1471	Cthe_1471	Glycoside hydrolase, family 5	−2	–0.29	–0.94	1.31	1.45E−02	C3
Cthe_1256	bglB	Glycoside hydrolase, family 3-like protein	1.5	3.17	2.46	2.17	2.11E−02	C4
Cthe_3063	Cthe_3063	Acetyl xylan esterase	–1.18	–1.36	–1.09	0.71	2.63E−02	C4
Cthe_2989	Cdp	Glycosyltransferase 36	2.95	3.61	3.42	3.29	3.87E−02	C4

The values at the different time points are log2 (NSAF) values

Among the 19 catalytic cellulosomal proteins in cluster 3 and 4, CelS, CelK, CtMan5A and CbhA were found to be most abundant and all classified under cluster 4. These enzymes provide a mixture of endo- and exo-glucanase activities to effectively access and process cellulose to smaller cellodextrins that can be imported and fermented [10, 40] and have been observed previously for cells using pure/semi-pure cellulose as the substrate [4, 15]. With regard to switchgrass-derived hemicellulose, the major constituent is xylan: a polymer of the C5 sugar xylose/arabinose. It is thus of interest to monitor the level of expressed xylanases over time. This is particularly relevant to industrial processes that would employ *C. thermocellum*, since the wild-type organism is unable to utilize/ferment xylans and/or C5 sugars, but requires their deconstruction for overall cellulose access [41, 42]. Note that hemicelluloses can also be inhibitory to cellulases, so it appears that there is a temporal balance between C5 and C6 deconstruction dynamics. Out of six detected xylanases, four β-xylanases (Cthe_0912, Cthe_1963, Cthe_2590, Cthe_2972) were classified under cluster 3 while Cthe_1398 (Xyloglucanase, Xgh74A) was classified under cluster 4. As mentioned above, both categories generally exhibit their highest abundance in stationary phase. The other xylanase, Cthe_1838, responsible for endohydrolysis of (1→4)-β-d-xylosidic linkages in xylans, did not change significantly over time. Considering the intertwined, heterogenous nature of lignocellulosic biomass, these xylanases play an important role in the general deconstruction of xylan-comprised hemicellulose encasing cellulose microfibrils, thus increasing the cellulase accessibility to the embedded cellulose [41, 42].

The general partitioning of xylanases to cluster 3 relative to other major cellulolytic components of the cellulosome (i.e., CipA, higher-order CipA-binding scaffoldins, and cellulases) to cluster 4 is particularly interesting. Xylanases other than Xgh74A rapidly accumulate from the start of the fermentation and increase consistently until stationary phase, whereas the cellulosome in general increases only modestly during log phase. This suggests an initial and immediate need for xylanase to deconstruct the hemicellulosic component of switchgrass and is perhaps critical to the initiation of switchgrass deconstruction.

### Inspection of key metabolic pathways for hydrolyzed cellulose utilization

**Fig. 3:**
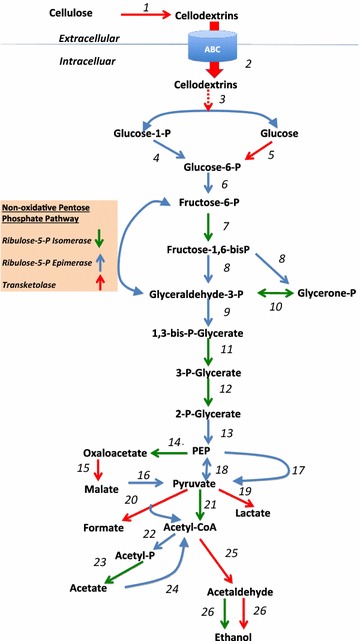
Glycolytic pathway. All the enzymes shown in the pathways were detected in mass spectrometry experiments. The *red arrow* in the pathway refers to the significant enzymes that belong to clusters 3 or 4. The *green arrow* refers to the significant enzymes that belong to clusters 1 and 2. The *blue arrow* refers to the enzymes that were detected in mass spectrometry but were not significantly changing over time. *1. Cellulosomes*; *2. Cellodextrin ABC transporters* Cthe_1019, 1020, 1862; *3. Cellodextrin phosphorylase* Cthe_2989; *4. Phosphoglucomutase* Cthe_1265; *5. Glucokinase* Cthe_0390; *6. Gluco*-*6*-*P*-*Isomerase* Cthe_0217; *7. Phosphofructokinase* Cthe_0347; *8. Fructose*-*bis*-*P*-*aldolase* Cthe_0349, 0319; *9. Glyceraldehyde*-*3*-*P*-*dehydrogenase* Cthe_0137; *10. Triose*-*phosphate isomerase* Cthe_0139; *11. Phosphoglycerate kinase* Cthe_0138; *12. Phosphoglycerate mutase* Cthe_0140; *13. Enolase* Cthe_0143; *14. PEP Carboxykinase* Cthe_2874; *15. Malate dehydrogenase* Cthe_0345; *16. Malic enzymes* Cthe_0344; *17. Pyruvate phosphate dikinase* Cthe_1308; *18. PEP synthase* Cthe_1253; *19. Lactate dehydrogenase* Cthe_1053; *20. Pyruvate formate lyase* Cthe_0506, 0505; *21. Pyruvate ferredoxin oxidoreductase* Cthe_2391, 2393; *22. Phosphotransacetylase* Cthe_1029; *23. Acetate kinase* Cthe_1028; *24. Acetyl CoA synthetase* Cthe_0551; *25. Aldehyde dehydrogenase* Cthe_0423* and *26. Alcohol dehydrogenase* Cthe_0423*, 0394, 2579 (*bifunctional enzymatic activity)

*Clostridium thermocellum* is known to utilize ABC transporter systems for active uptake of oligosaccharides generated during cellulose hydrolysis [43]. Out of seven detected cellodextrin ABC transporter components, three (Cthe_1862, Cthe_1020, and Cthe_1019) were classified into cluster 4, whereas Cthe_0393 was a member of cluster 3. This suggests the majority of proteins required for the cellodextrin uptake remain relatively constant, with a modest increase at the transition between growth phases, similar to what was observed for CipA and other major cellulosome components. A transcriptomic analysis of *C. thermocellum* grown on pure cellulose showed similar findings with regard to cellodextrin transport [13]. As shown in Fig. 3 (Additional file 9: Fig. S2), the transported cellodextrins are further processed to glucose-1-phosphate and glucose in presence of cellodextrin phosphorylase, Cthe_2989, a member of cluster 4. Glucose-1-phosphate is further converted to the glycolytic intermediate glucose-6-phosphate in the presence of phosphoglucomutase (Cthe_1265), whereas glucose converts to glucose-6-phosphate in presence of glucokinase, Cthe_0390, a member of cluster 4.

When the abundances of enzymes involved in the glycolytic pathway were assessed (Fig. 3), those involved in the conversion of glucose-6-phosphate to phosphoenol pyruvate (PEP) either had no change in expression (blue arrows) or showed decreased abundance over time (green arrows). This would be expected as the organism enters stationary phase and corroborates other studies [13]. Although glycolytic pathway enzyme abundance is stable/reduced, there are several metabolic outcomes that are enhanced as cells leave exponential growth, namely the interconversion of oxaloacetate ⇔ malate (malate shunt) and the production of ethanol and organic acids of anaerobic fermentation. These processes are discussed below.

While several studies [35, 44] have suggested that pyruvate phosphate dikinase (PPDK; Cthe_1308) does not play a crucial role for the generation of pyruvate directly from phosphoenolpyruvate (PEP), our study finds a high abundance of PPDK that was constant throughout the time course (Fig. 3). However, *C. thermocellum* normally forms pyruvate via a malate shunt, which includes PEP carboxykinase (Cthe_2874; cluster 2), malate dehydrogenase (Cthe_0345; cluster 4), and malic enzyme (Cthe_0344; no change) [44]. It has been suggested that the organism diverts carbon flow through the shunt to increase the production of the biosynthetic intermediates NADPH and GTP [35]. All three enzymes were detected at high abundance, but had varied abundance trends.

The resulting pyruvate is the precursor for all fermentative endpoints. Examination of protein abundances profiles revealed that, except for acetate, all other end product-related enzymes appeared to increase over time, supporting the observed accumulation of these end products via microbial metabolism. These include lactate dehydrogenase (Cthe_1053; cluster 3) catalyzing pyruvate → lactate, pyruvate formate lyase (Cthe_0505 and Cthe_0506; cluster 4) catalyzing pyruvate → formate, and AdhE (Cthe_0423; cluster 4), a bidirectional acetaldehyde/CoA dehydrogenase. This enzyme functions as both an alcohol dehydrogenase and acetaldehyde dehydrogenase and is the major driver of ethanol production in *C. thermocellum* and other ethanologenic microbes [14, 45, 46]. These results suggest that fermentation continues as long as there is available glucose and even into stationary phase, which is in line with other studies [47].

### Induction of pentose phosphate pathway towards late-stage culture

**Fig. 4:**
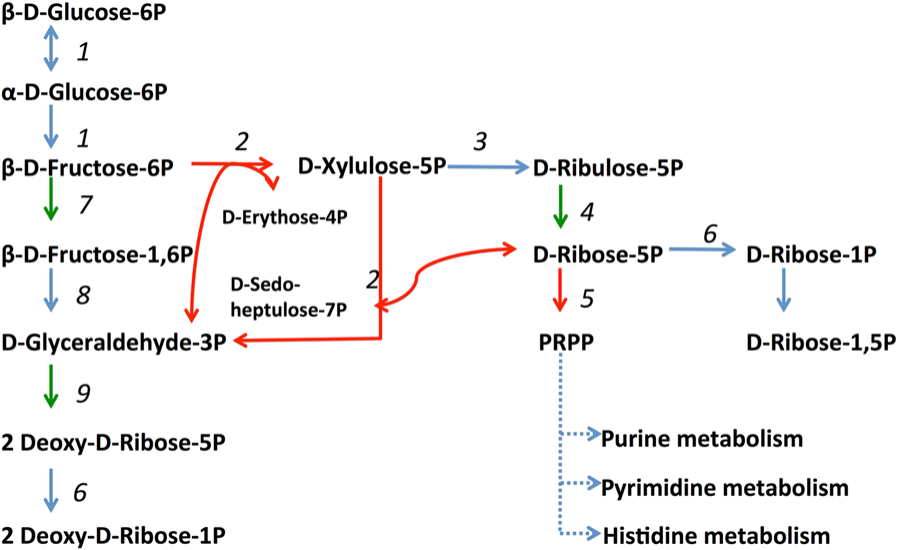
Pentose phosphate pathway. All the enzymes required were detected in mass spectrometry experiments. The *red arrow* in the pathway refers to the significant enzymes that belong to clusters 3 or 4. The *green arrow* refers to the significant enzymes that belong to clusters 1 and 2. The *blue arrow* refers to the enzymes that were detected in mass spectrometry but were not significantly changing over time. *1. Glucose*-*6*-*phosphate isomerase* Cthe_0217; *2. Transketolase subunit A* Cthe_2443, 2704; *3. Ribulose*-*5*-*phosphate 3*-*epimerase* Cthe_0576; *4. Ribose*-*5*-*phosphate 3*-*isomerase* Cthe_2597; *5. Ribose*-*phosphate pyrophosphokinase* Cthe_2630; *6. Phosphopentomutase* Cthe_0677; *Phosphoglucomutase* Cthe_1265; *7. 6*-*Phosphofructokinase* Cthe_0347; *8. Fructose*-*bisphosphate aldolase* Cthe_0349 and *9. Deoxyribose*-*phosphate aldolase* Cthe_1943

Even though glycolysis is the primary metabolic pathway employed by anaerobes for energy generation, we investigated how the organism may shift glycolytic intermediates to alternate pathways. This might be a useful adaptation to overcome metabolic inhibition. An example of this was observed for the pentose phosphate pathway (PPP), which utilizes glycolytic intermediates to produce pentose (C5) sugars required for the biosynthesis of nucleotides and aromatic amino acids [13]. Out of 14 total detected PPP-related enzymes, six significantly changed with time (Fig. 4). Of these, transketolases (Cthe_2443, 2704) and ribose-phosphate pyrophosphokinase (Cthe_2630), members of cluster 4, were found to increase upon entry into early stationary phase. The high abundance and increasing accumulation of ribose-phosphate pyrophosphokinase suggests production of phosphoribosyl pyrophosphate (PRPP), a sugar precursor for purine and pyrimidine metabolism. As this portion of the PPP is generally up-regulated toward the end of the culture, where growth-related demand for nucleotides and aromatic amino acids is likely subsiding, diversion to this pathway could provide a vital shunt to divert carbon/electrons away from glycolysis in an attempt to balance metabolic flux.

With regard to the other C5 sugar-related enzymes, we also detected a very high abundance of alcohol dehydrogenase GroES-like protein (Cthe_0388; cluster 2). This enzyme converts D-xylulose (hemicellulose-derived sugar) into xylitol, which accumulated almost ninefold in late-stationary phase (Additional file 2: Table S1). Even though the abundance of this protein decreases significantly with time, it is still highly abundant during late-stationary phase and likely still involved in the interconversion of xylulose/xylitol. However, as *C. thermocellum* lacks an annotated xylose reductase, the only way xylitol can be formed biologically is by the reduction of xylulose, potentially by Cthe_0388 enzyme mentioned above. Further work is needed to confirm this additional functionality. Compared to xylitol, xylulose increased only ~2-fold by stationary phase. There are two possibilities for this accumulation; xylulose could be a direct product from hemicellulose degradation or there is an unknown phosphate transferase that converts xylulose-5-phosphate to/from xylulose. This could be of potential value to industrial fermentation, as xylitol is used as artificial sweetener and antimicrobial agent in foods [48, 49].

### Branched-chain amino acid pools serve as precursors for higher-order fatty acid accumulation

**Fig. 5:**
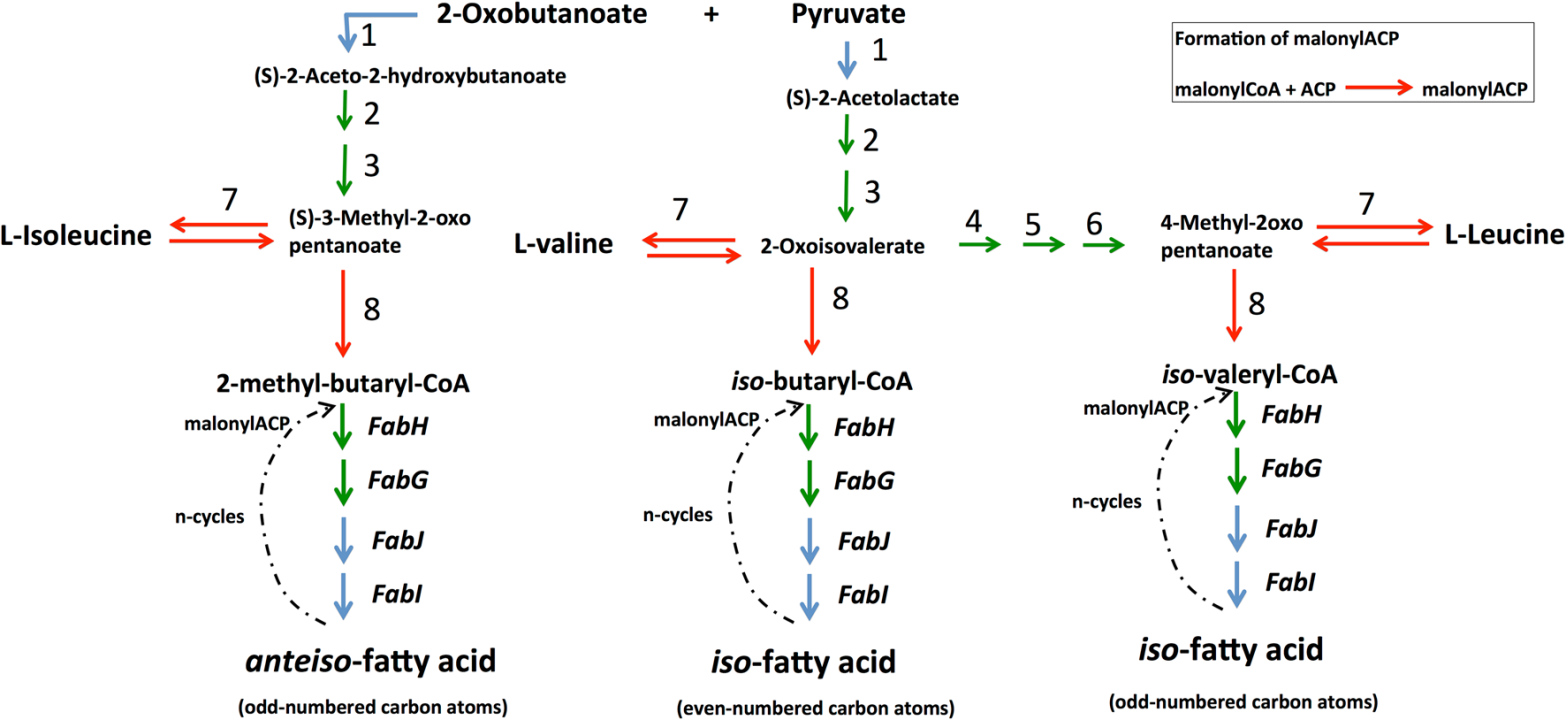
Valine, leucine, and isoleucine yield precursors for branched-chain fatty acid synthesis The *red arrow* in the pathway refers to the significant enzymes that belong to clusters 3 or 4. The *green arrow* refers to the significant enzymes that belong to clusters 1 and 2. The *blue arrow* refers to the enzymes that were detected in mass spectrometry but were not significantly changing over time. *1. Acetolactate synthase* Cthe_2516, 2517, 2714; *2. Ketol*-*acid reductoisomerase* Cthe_2518; *3. Dihydroxy*-*acid dehydratase* Cthe_2713; *4. 2*-*Isopropylmalate synthase* Cthe_1391, 2519; *5. 3*-*Isopropylmalate dehydratase* Cthe_2210, 2211; *6. 3*-*Isopropylmalate dehydrogenase* Cthe_2209; *7. Branched-chain amino acid aminotransferase* Cthe_0856; *8. Branched-chain keto acid dehydrogenase* Cthe_0547*—possible new functional annotation [52]—Fatty acid synthase enzymes (Fab H,G,J,I reported in Additional File 10: Table S7

Aside from its ultimate fermentation to ethanol and organic acids, metabolic outcomes of PEP/Pyruvate as an intermediate include the biosynthesis of the branched-chain amino acids (BCAA) valine, leucine, and isoleucine. All three amino acids accumulated—each rising to their maximum (or close to it) by 43 h of growth and remaining elevated, relative to 19 h (Additional file 2: Table S1). Leucine accumulation was most dramatic (~10-fold), followed by isoleucine (~5-fold), and valine (~1.5-fold). Although protein machinery involved in the conversion of pyruvate to BCAAs generally did not change or decreased over time (Fig. 5), a BCAA aminotransferase (Cther_0856) controlling the final step of valine, leucine, and isoleucine biosynthesis (or their degradation) increased significantly (cluster 4), suggesting an important role for these BCAAs or their derivatives.

One possibility is that *C. thermocellum* shifts its metabolism toward the biosynthesis of amino acids utilizing PEP/pyruvate as the precursor. This would be one of the several ways to rebalance metabolism. Diversion of carbon flux to amino acid production has been previously documented in *C. thermocellum* grown on model cellulose substrates like cellobiose [50] or pure cellulose [47]. Although increased amino acid production was striking in these earlier studies, growth on pretreated switchgrass did not elicit the same robust response as observed with the model substrates. This was true for both intracellular and supernatant metabolite pools (data not shown). In fact, the production of amino acids due to overflow metabolism in late-stage cultures was relatively muted, appearing to preferentially affect only the BCAAs described above.

Considering the relative ratios of BCAAs observed here, especially when compared to other studies utilizing model cellulosic substrates, we report another possibility linked to the production of BCAAs that involves synthesis of higher-order fatty acids. Degradation of all three BCAAs, ultimately to isobutyryl-CoA (valine), isovaleryl-CoA (leucine), and 2-methyl-butyryl-CoA (isoleucine), gives rise to anteiso/odd, iso/odd, and iso/even type fatty acids [51]. This could explain the large accumulation of these type of fatty acids revealed by metabolomic analysis. As shown in Fig. 5, the first step in BCAA degradation pathway requires the same up-regulated BCAA aminotransferase (Cthe_0856) involved in their synthesis. However, to obtain the activated, higher-order fatty acid precursors listed above, a second enzymatic step is required. To this end, a recent metabolic analysis of *C. thermocellum* has suggested a new functional annotation for the enzyme encoded by Cthe_0547 that would fulfill this role—a branched-chain keto acid dehydrogenase [52]. This enzyme would be able to convert 2-oxoisovalerate (from valine), 4-methyl-2-oxopentanoate (from leucine), and 3-methyl-2-oxopentanoate (from isoleucine) to isobutyryl-CoA, isovaleryl-CoA, and 2-methyl-butyryl-CoA, respectively—each providing the necessary building blocks for the production of these higher-order fatty acids [51]. Encouragingly, Cthe_0547 was found to significantly increase with time (cluster 3) providing enzymatic evidence for the measured metabolic accumulation of these types of fatty acids over the course of growth.

### Fatty acid biosynthesis as a fermentation response

Bacterial fatty acid production primarily contributes to the formation and maintenance of their lipid membranes [53]. Accumulation of specific types of fatty acids would provide a mechanism for *C. thermocellum* to adapt to either the build-up of fermentation products and/or lignocellulose-derived inhibitory compounds. However, as the level of ethanol and other fermentation products were not high enough to be considered inhibitory, the latter seems to be a more reasonable explanation. This is further substantiated by the accrual of lignocellulose-derived inhibitory compounds, including 4-hydroxybenzoic acid, vanillic acid, ferulic acid, *p*-coumaric acid, and vanillin (fold change range 2.2–1.8x), during *C. thermocellum* growth on switchgrass. As the chain length, saturation, and branching status of fatty acids generally control the fluidity/rigidity of the membrane, many bacteria adapt to inhibitors such as these by changing the fatty acid composition of their membrane lipids [32, 33].

Our results indicate that *C. thermocellum* increases the production of long-chain, branched iso- and anteiso-fatty acids over time, perhaps in response to the adverse effect imposed by lignocellulose-derived inhibitors and fermentation end products. The maintenance of optimal membrane fluidity/rigidity with branched-chain fatty acids depends on the availability of valine (anteiso/odd), leucine (iso/odd), and isoleucine (iso/even) [33]. The metabolite abundance profiles for both the BCAAs and higher-order fatty acids measured here (Additional file 2: Table S1) indicate a substantial increase in the amount of iso-fatty acids relative to their anteiso-counterparts. This corroborates a previous study where Gram-positive bacterium *Arthrobacter chlorophenolicus* exposed to toxic concentrations of phenol, 4-chlorophenol, and 4-nitrophenol led to a decrease in the anteiso/iso ratio; a response that made a more rigid membrane to counteract the increase in fluidity brought upon by the cytotoxic phenolics [54].

All necessary enzymes illustrated in the KEGG pathway for the conversion of acetyl-CoA into palmitic acid (base fatty acid) were detected in the *C. thermocellum* (Additional file 10: Table S7). Overall, enzymes involved in fatty acid biosynthesis were either stable or decreased over time, again following general cessation of growth. Interestingly, the reductive steps in the biosynthesis of fatty acids require NADPH, a major product of malate shunt pathway shown to be up-regulated above (Fig. 3). In the context of higher-order, odd numbered fatty acids, a three-carbon precursor, propionyl-CoA, is required rather than acetyl-CoA [55]. Formate acetyl transferase (Cthe_0505) is capable of producing propionyl-CoA from 2-oxobutanoate, a product formed from cysteine and methionine metabolism. This enzyme was found to increase over time (cluster 4). Together with the increase of higher-order, long-chain fatty acids observed by metabolomics analysis, these data suggest that although the base machinery for producing fatty acids decreases toward late-stage cultures as overall metabolic demand tapers off, enzymes responsible for branched, odd numbered fatty acid precursors are still required (and up-regulated) for overall membrane maintenance and/or continued turnover. This implies that the deconstruction of switchgrass imposes greater metabolic challenges on *C. thermocellum* than do model cellulose substrates, spurring the organism to adapt its cellular membrane to the increasingly hostile culture environment toward late-stage cultures.

## Conclusions

*Clostridium thermocellum* is a candidate for consolidated bioprocessing of cellulosic biomass to biofuels. In order to understand the delicate balance between recalcitrant lignocellulose solubilization, end product formation, and potential microbial inhibition, we have conducted a detailed *C. thermocellum* time course omics analysis to characterize its growth on industrially relevant, dilute acid pretreated switchgrass. As expected, we found that most central metabolism-related enzymes decreased in abundance with growth, suggesting cessation of biogenesis-related activity towards late-stationary phase. In contrast, the level of enzymes involved in the production of fermentation end products remained high, potentially triggering the up-regulation of carbon/electrons diverting into metabolic pathways such as PPP to maintain metabolic flux. An important finding from this study was the observation that hemicellulose-derived 5C sugars/sugar alcohols and phenolic acids derived from lignin accumulated over time and likely contributed to growth inhibition, thus prompting up-regulation of BCAA/fatty acid re-composition pathways to adapt the cell to deteriorating culture conditions brought upon by continued deconstruction of switchgrass. This is further substantiated by the observed accumulation of higher-order fatty acids synthesized by *C. thermocellum*. In total, the time-dependent accumulation of long-chain iso- (and less so anteiso-) over linear fatty acids, as well as the corresponding up-regulation of the required biosynthetic protein machinery, suggests a protective effect that is imparted by these higher-order, long-chained fatty acids and is likely a necessary metabolic adaptation to growth on complex lignocellulosic biomass.

## Additional files

**Additional file 1: Text S1.** Supplemental materials and methods description.

**Additional file 2: Table S1.** Details of metabolites detected in the time course analysis of *C. thermocellum* grown on switchgrass.

**Additional file 3: Table S2.** Normalized spectral abundance factor (NSAF) normalized protein spectral count measurements using mass spectrometry.

**Additional file 4: Table S3.** Global proteomic metrics.

**Additional file 5: Table S4.** Expression level of transcripts for *C. thermocellum* grown on switchgrass at 19 and 43 h time points.

**Additional file 6: Table S5.** The comparison table of position preference, gene expression and MS detected proteins.

**Additional file 7: Table S6.** Significantly time-dependent (*p* < 0.05) proteins grouping into four different clusters.

**Additional file 8: Fig. S1.** Temporal expression based clustering of proteins differentially expressed during switchgrass fermentation.

**Additional file 9: Fig. S2.** Heatmap showing the differentially abundant enzymes involved in the glycolytic pathway.

**Additional file 10: Table S7.** Enzymes detected for fatty acid biosynthesis in *C. thermocellum.*

## Abbreviations

C5: 5-carbon sugar
C6: 6-carbon sugar
CBP: consolidated bioprocessing
CAZymes: carbohydrate-active enzymes
LC–MS/MS: liquid chromatography–tandem mass spectrometry
GC–MS: gas chromatography mass spectrometry
DTT: dithiothreitol
MudPIT: multidimensional protein identification technology
LTQ: linear trapping quadrupole
MS/MS: tandem mass spectrometry
SpC: spectral count
NSAF: normalized spectral abundance factors
ANOVA: analysis of variance
COG: clusters of orthologous groups
KEGG: Kyoto Encyclopedia of Genes and Genomes
GO: gene ontology
ATCC: American Type Culture Collection
MS: mass spectrometry/mass spectrometer
BCAA: branched-chain amino acids
