# Supplementary material for: Integrated omics analyses reveal the details of metabolic adaptation of Clostridium thermocellum to lignocellulose-derived growth inhibitors released during the deconstruction of switchgrass
Source: Biotechnol Biofuels. 2017 Jan 10;10:14. doi: 10.1186/s13068-016-0697-5 (PMC5223564; doi:10.1186/s13068-016-0697-5)
Supplement: Supplementary file 1 — Additional file 1: Text S1. Supplemental materials and methods description. [file 13068_2016_697_MOESM1_ESM.docx]

**Integrated ‘omics analyses reveals the details of metabolic adaptation of *Clostridium thermocellum* to lignocellulose-derived growth inhibitors released during the deconstruction of switchgrass.**

^†^ Suresh Poudel^1,3^; Richard J. Giannone^2^; Miguel Rodriguez Jr.^1^; Babu Raman^4^; Madhavi Z. Martin^1^; Nancy L. Engle^1^; Jonathan R. Mielenz^1^; Intawat Nookaew^1,5^; Steve D. Brown^1^; Timothy J. Tschaplinski^1,3^; David Ussery^1,5^; and Robert L. Hettich^2,3^*

^1^Biosciences Division; ^2^Chemical Sciences Division; Oak Ridge, TN 37831, USA ^3^Department of Genome Science and Technology, University of Tennessee, Knoxville, TN 37996,^4^Dow AgroSciences, 9330 Zionsville Road, Indianapolis, IN 46268 USA.^5^Present address: Department of Biomedical Informatics, University of Arkansas for Medical Sciences, AR 72205.

**Supplemental Materials and Methods S1**

**Cultivation and sampling.** The main triplicate fermentations were inoculated from a pH controlled seed fermenter culture, which was grown for 125 h and contained switchgrass as the substrate. The seed vessel was inoculated from exponential growth stage cells cultured overnight in serum bottles with MTC media and Avicel PH-105 (FMC BioPolymer, Newark, DE, USA) as the carbon source. Base addition plots were used as a guide to determine the growth stage of the cells in the fermenters. Fermentation products and residual carbohydrates were analyzed from samples taken throughout the fermentations, as previously described [1].

**Metabolomic measurements.** Metabolite peaks were extracted using a key selected ion, characteristic m/z fragment, rather than the total ion chromatogram, to minimize integrating co-eluting metabolites. The extracted peaks of known metabolites were scaled back up to the total ion current using predetermined scaling factors. Peaks were quantified by area integration and the concentrations normalized to the quantity of the internal standard recovered, amount of sample extracted, derivatized, and injected. A large user-created database was used to identify the metabolites of interest to be quantified. The database consisted of ~2300 spectra, of mass spectral electron impact ionization (EI) fragmentation patterns of TMS-derivatized compounds, as well as the Wiley Registry 10th Edition combined with NIST 2014 mass spectral library. There were three replicates per sampling time point.

**Proteomic measurements.** The resulting crude lysates were incubated at 60˚C for 1 hour. Denatured and reduced protein samples were then diluted to 1 M guanidine-HCl with 50 mM Tris-HCl, pH 8.0 and digested with two sequential aliquots of sequencing-grade trypsin (Promega Corp., Madison, WI, USA) at a 1:100 enzyme:protein ratio (w/w), initially overnight and then followed by 4 h. Following digestion, each sample was desalted and solvent exchanged to acidified water (0.1% formic acid) via Sep-Pak (Waters, Milford, MA, USA) as previously described [2]. Resulting peptide concentrations were measured using the BCA protein assay kit.

Aliquots of 100 micrograms of proteolytic peptides were bomb-loaded onto a biphasic MudPIT back column as previously described [3, 4]. Loaded peptides were then placed in-line with an in-house pulled, reverse-phase packed nanospray emitter and analyzed by a 12-step MudPIT over the course of 24 hrs, with each step consisting of a salt-pulse of increasing concentration of ammonium acetate (up to 500 mM), each followed by a 2-hr reverse phase gradient from solvent A (95% water, 5% acetonitrile, 0.1% formic acid) to 50% solvent B (30% water, 70% acetonitrile, 0.1% formic acid). LC-resolved peptides were analyzed in real-time by an LTQ-XL mass spectrometer (ThermoFisher Scientific, Grand Island, NY, USA) operating in a data-dependent fashion as previously described [2, 5]. A total of two replicate measurements were obtained for each of three biological replicates per time point for a total of 24 LC-MS/MS measurements.

Acquired MS/MS spectra were assigned to specific peptide sequences using SEQUEST[6] with a FASTA proteome database consisting of the *C. thermocellum* 27405 genome (version CP000568.1 GI:125712750) [1] concatenated with common contaminants as well as reversed decoy sequences to assess protein-level false discovery rates. SEQUEST-scored peptide sequence data were filtered and assembled into protein loci using DTASelect [7] with the following conservative criteria: XCorr: +1 = 1.8, +2 = 2.5, +3 = 3.5, DeltCN 0.08, and 2 peptides per protein identification with at least one required to be unique.

**Transcriptome measurements.** RNA was isolated using the TRIzol reagent (Invitrogen, Carlsbad, CA) with bead-beating and purified using the Qiagen RNeasy Mini kit in accordance with the instructions from the manufacturer and included a column based Qiagen DNase treatment [1]. Total cellular RNA was quantified with a NanoDrop ND-1000 spectrophotometer (NanoDrop Technologies, DE) and RNA quality was assessed with Agilent Bioanalyzer (Agilent Technologies Inc. CA). Template cDNA preparation, sample labeling with Cy3-dye, and DNA microarray hybridizations were conducted following the NimbleGen protocols and as described previously [1], except that high density Nimblegen tiling arrays were used in this study. Microarray data and platform details have been deposited in the NCBI Gene Expression Omnibus (GEO) database under accession number GSE26926, with data used in this study having accession numbers GSM663002-GSM663007. Transcriptomics data underwent LOESS normalization and a Student *t- test* was conducted to compare data from the two time points. The up-regulated and down-regulated genes were determined by using a threshold of 2X fold change and a p-value < 0.05.

The CMG-Biotools package [8] was used to create a genome atlas of *C. thermocellum* ATCC 27405. This genome atlas is circular plot that contains DNA structural information mapped along the chromosome (percent AT and GC skew, DNA repeats, stacking energy, intrinsic curvature and position preference) [9, 10]. The DNA sequence was read and an output file was created that generated numerical number ‘position preference’ that corresponds to each nucleotide in the genome [8]. The ‘position preference’ measure of the chromosomal DNA sequence was used to predict highly expressed regions in the DNA, as described previously [11]. Briefly, using a file with the numerical value (position preference) generated for each tri-nucleotide along the chromosome, a file was generated, with an average value at each position reflecting a 7.6 kbp window. Regions of at least 7.6 kbp (genome length * 0.002)[8], with more than 2 standard deviations less than the chromosomal average were extracted with a python code. Genes within these regions had the lowest position preference value (< 2 SD) from the average value and thus were termed as ‘predicted highly expressed genes’. The transcriptome and proteome information were mapped to the chromosome, and given an intensity related to abundance (log2 NSAF), and the lanes were added to the blast atlas.

**References**:

1. Wilson CM, Rodriguez CM, Johnson SL, Martin T, Chu RD, Wolfinger LJ, Hauser ML, Land DM, Klingeman MH, Syed AJ *et al*: **Global transcriptome analysis of Clostridium thermocellum ATCC 27405 during growth on dilute acid pretreated Populus and switchgrass**. *Biotechnology for biofuels* 2013, **6**(1):179.

2. Lochner A, Giannone RJ, Keller M, Antranikian G, Graham DE, Hettich RL: **Label-free quantitative proteomics for the extremely thermophilic bacterium Caldicellulosiruptor obsidiansis reveal distinct abundance patterns upon growth on cellobiose, crystalline cellulose, and switchgrass**. *Journal of proteome research* 2011, **10**(12):5302-5314.

3. Washburn MP, Wolters D, Yates JR: **Large-scale analysis of the yeast proteome by multidimensional protein identification technology**. *Nat Biotechnol* 2001, **19**(3):242-247.

4. McDonald WH, Ohi R, Miyamoto DT, Mitchison TJ, Yates JR: **Comparison of three directly coupled HPLC MS/MS strategies for identification of proteins from complex mixtures: single-dimension LC-MS/MS, 2-phase MudPIT, and 3-phase MudPIT**. *Int J Mass Spectrom* 2002, **219**(1):245-251.

5. Lochner A, Giannone RJ, Rodriguez M, Shah MB, Mielenz JR, Keller M, Antranikian G, Graham DE, Hettich RL: **Use of label-free quantitative proteomics to distinguish the secreted cellulolytic systems of Caldicellulosiruptor bescii and Caldicellulosiruptor obsidiansis**. *Applied and environmental microbiology* 2011, **77**(12):4042-4054.

6. Eng JK, McCormack AL, Yates JR: **An approach to correlate tandem mass-spectral data of peptides with amino-acid-sequences in a protein database**. *J Am Soc Mass Spectrom* 1994, **5**(11):976-989.

7. Tabb DL, McDonald WH, Yates JR: **DTASelect and contrast: Tools for assembling and comparing protein identifications from shotgun proteomics**. *J Proteome Res* 2002, **1**(1):21-26.

8. Vesth T, Lagesen K, Acar O, Ussery D: **CMG-biotools, a free workbench for basic comparative microbial genomics**. *PloS one* 2013, **8**(4):e60120.

9. Jensen LJ, Friis C, Ussery DW: **Three views of microbial genomes**. *Research in Microbiology* 1999, **150**(9–10):773-777.

10. Pedersen AG, Jensen LJ, Brunak S, Stærfeldt H-H, Ussery DW: **A DNA structural atlas for Escherichia coli**. *Journal of molecular biology* 2000, **299**(4):907-930.

11. Willenbrock H, Ussery DW: **Prediction of highly expressed genes in microbes based on chromatin accessibility**. *BMC molecular biology* 2007, **8**(1):11.
