# Supplementary material for: Integrated omics analyses reveal the details of metabolic adaptation of Clostridium thermocellum to lignocellulose-derived growth inhibitors released during the deconstruction of switchgrass
Source: Biotechnol Biofuels. 2017 Jan 10;10:14. doi: 10.1186/s13068-016-0697-5 (PMC5223564; doi:10.1186/s13068-016-0697-5)
Supplement: Supplementary file 4 — Additional file 4: Table S3. Global proteomic metrics. [file 13068_2016_697_MOESM4_ESM.docx]

| TABLE S3. Global proteomic metrics | | |  |
| --- | --- | --- | --- |
| Type | Detected Proteins | Known Proteins | Missing |
| Ribosomal | 51 (small 21, large 32) | 53* | 2 (S23, L34) |
| ATP synthase | 12 | 14* | **2 (F0 sector subunit a, F1 sector epsilon subunit )** |
| Extracellular | 170 | ~300 | ~40% |
| Cellulosomal | 59 | 80+ | 20+ |
| Total | 1551 | 3107* | ~50% |

*** Data obtained from UniProtKB**
