# Supplementary material for: Integrated omics analyses reveal the details of metabolic adaptation of Clostridium thermocellum to lignocellulose-derived growth inhibitors released during the deconstruction of switchgrass
Source: Biotechnol Biofuels. 2017 Jan 10;10:14. doi: 10.1186/s13068-016-0697-5 (PMC5223564; doi:10.1186/s13068-016-0697-5)
Supplement: Supplementary file 8 — Additional file 8: Fig. S1. Temporal expression based clustering of proteins differentially expressed during switchgrass fermentation. [file 13068_2016_697_MOESM8_ESM.docx]

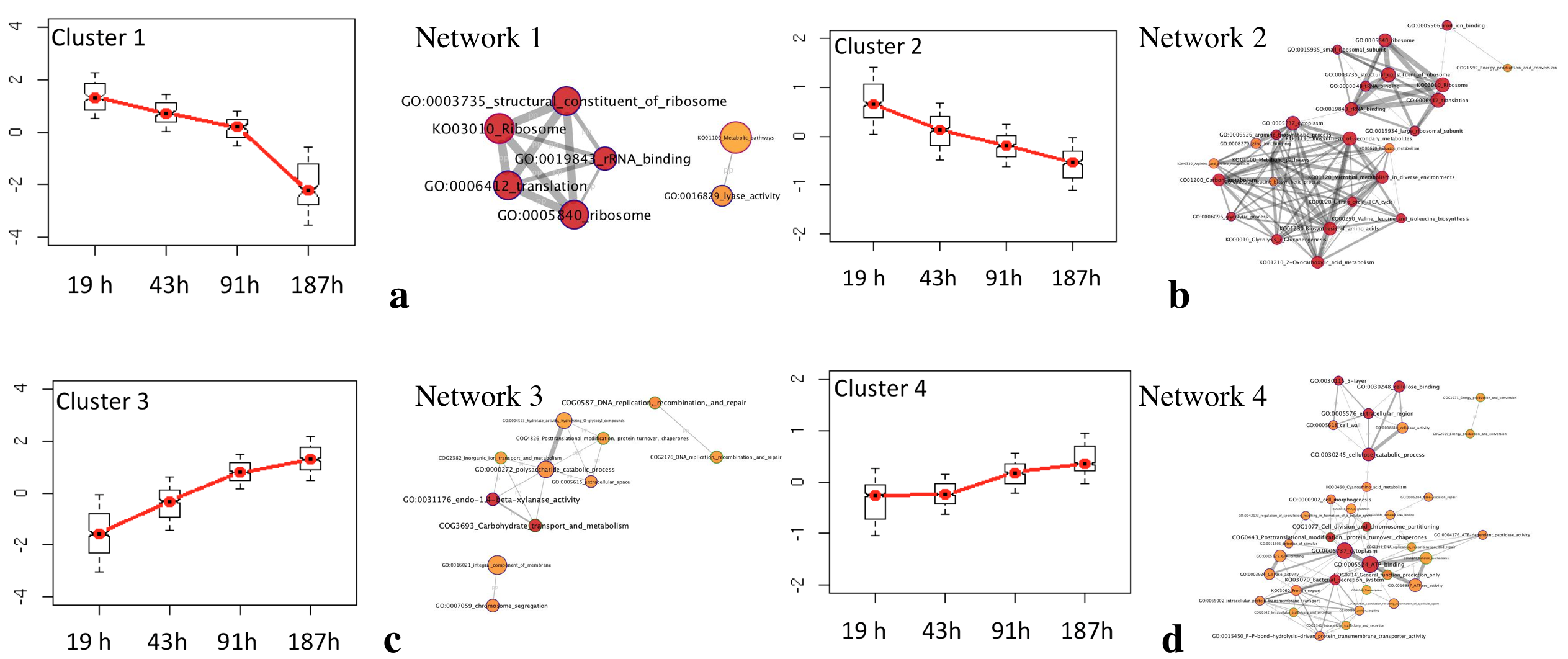


Fig. S1. **Temporal expression based clustering of proteins differentially expressed during switchgrass fermentation.** X-axis (Time Points) and Y-axis (Z-scores of spectral count) and **Network of clusters 1, 2, 3 and 4 respectively.** A cut-off P-value < 0.01 was used to build the network. Each node shows an enriched GO, KEGG or COG functional category. The node color and size represents the P-value and number of members for each node respectively. Darker red color represents lower P-value. The node size grows proportionally with the number of members assigned to a particular function. The predominant functional members of Network 1 represent ribosomes, Network 2 represents central carbon metabolism and ribosomal constituents, Network 3 represents carbohydrate transport and beta xylanase activity and Network 4 represents bacterial secretion system, ATP binding and cytoplasm.
