# Supplementary material for: Integrated omics analyses reveal the details of metabolic adaptation of Clostridium thermocellum to lignocellulose-derived growth inhibitors released during the deconstruction of switchgrass
Source: Biotechnol Biofuels. 2017 Jan 10;10:14. doi: 10.1186/s13068-016-0697-5 (PMC5223564; doi:10.1186/s13068-016-0697-5)
Supplement: Supplementary file 9 — Additional file 9: Fig. S2. Heatmap showing the differentially abundant enzymes involved in the glycolytic pathway. [file 13068_2016_697_MOESM9_ESM.docx]

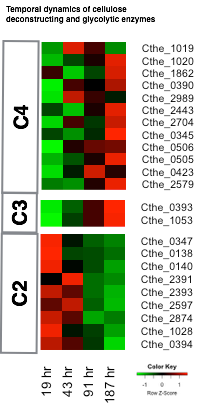


**Fig. S2. Heatmap showing the differentially abundant enzymes involved in the glycolytic pathway. (locus tag protein name mentioned in Fig. 3 main text)**
