# Supplementary material for: Integrated omics analyses reveal the details of metabolic adaptation of Clostridium thermocellum to lignocellulose-derived growth inhibitors released during the deconstruction of switchgrass
Source: Biotechnol Biofuels. 2017 Jan 10;10:14. doi: 10.1186/s13068-016-0697-5 (PMC5223564; doi:10.1186/s13068-016-0697-5)
Supplement: Supplementary file 10 — Additional file 10: Table S7. Enzymes detected for fatty acid biosynthesis in C. thermocellum. [file 13068_2016_697_MOESM10_ESM.docx]

| **Table S7. Enzymes detected for fatty acid biosynthesis in *C. thermocellum*** | | | |
| --- | --- | --- | --- |
| **Gene** | **Protein** | **Locus tag** | **Cluster** |
| FabD | [Acyl-carrier-protein] S-malonyltransferase | Cthe_0935 | C2 |
| FabH | 3-oxoacyl-(acyl-carrier-protein) synthase | Cthe_0130 | C4 |
|  |  | Cthe_0132 |  |
|  |  | Cthe_0936 | C2 |
| FabF | 3-oxoacyl-[acyl-carrier-protein] synthase II | Cthe_0932 | C2 |
| FabG | 3-oxoacyl-[acyl-carrier-protein] reductase | Cthe_0934 |  |
| FabZ | (3R)-hydroxymyristoyl-ACP dehydratase | Cthe_2625 |  |
| FabI | enoyl-[acyl-carrier-protein] reductase | Cthe_3169 |  |
|  | AMP-dependent synthetase and ligase | Cthe_1232 |  |
